# One-year anniversary: The progress of *Microsystems & Nanoengineering*

**DOI:** 10.1038/micronano.2016.46

**Published:** 2016-07-25

**Authors:** Yirong Wu, Ian White, Tianhong Cui

**Affiliations:** 1 Editor-in-Chief, *Microsystems & Nanoengineering*; Academician of Chinese Academy of Sciences and Director of Institute of Electronics, Chinese Academy of Sciences, Beijing, China; 2 Executive Editor-in-Chief, *Microsystems & Nanoengineering*; Fellow of Royal Academy of Engineering and Professor, University of Cambridge, Cambridge, UK; 3 Executive Editor-in-Chief, *Microsystems & Nanoengineering*; Professor, University of Minnesota, 111 Church Street S.E., Minneapolis, MN 55455, USA

With time passing imperceptibly, *Microsystems & Nanoengineering* has indeed attracted the attention of its international peers in MEMS and nanotechnology since it was launched as a new journal on May 28, 2015. *Microsystems & Nanoengineering* is the first engineering journal co-published by the Nature Publishing Group (NPG) and the Institute of Electronics of Chinese Academy of Sciences (IECAS). On behalf of the editorial board of *Microsystems & Nanoengineering*, we would like to express our deep gratitude to our readers, authors, and reviewers. We sincerely thank all of you for your strong and consistent support!

As we all know, for an international journal to have great impact globally, its publications must be of the highest quality and reflect the research field globally. Only by receiving original papers from around the world and assessing them by international peers, we can select the high-quality papers to match the leading academic standards. The quality of the review and processing of manuscripts is essential for us.

On this basis, since launching the journal, *Microsystems & Nanoengineering* has been adhering to the rigorous peer-review process. As it was founded last year, with the assistance of our international editorial board members and editors from around the world as well as benefitting from the international profile of the NPG, *Microsystems & Nanoengineering* has accumulated experts from 22 countries and regions to submit 110 invited and contributed papers on their most recent research (Figure 1). Using our strict and rigorous review process, 48 peer-reviewed papers from 69 academic organizations in 13 countries and regions were formally accepted for publication (Figure 2). Several leading experts were invited as new editorial board members, and now the editorial board has 40 internationally renowned experts from 12 countries and regions (Figure 3).

The international peer-review process can ensure the quality of publications to meet the international standards at a high level. Currently, the reviewer database of *Microsystems & Nanoengineering* has more than 3000 experts. As of May 28, 2016, 202 international experts from 27 countries and regions are involved in the peer-review process of submitted manuscripts (Figure 4). Hereby we express our heartfelt thanks for their professional service and strong support. *Microsystems & Nanoengineering* constantly strives to facilitate and stimulate the research community to develop new concepts and methods for advancement of some specific hot topics. Three special issues including Micro and Nano Systems for Cell Analysis, Nanomanufacturing, Optofluidics are under development, and are scheduled for publications starting from the end of 2016.

The journal aims to publish leading, peer-reviewed fundamental and applied research in the exciting and emerging fields of microsystems and nanoengineering. Submissions are welcome in fields including design (theory, modeling, or simulation), fabrication, characterization, reliability, packaging, and applications of devices and systems with micro- or nano-scale features. This new joint publication will provide a rapid forum for the dissemination of original research articles and selected high-quality review articles in all aspects of microsystems and nanoengineering with an emphasis on fundamental research. We will continue to publish papers alongside more general research summaries to increase readership and create impact, in a manner that will distinguish the new *Nature* journal from other journals in MEMS and nanoengineering.

Benefitting from the great publication expertise of both the NPG and the IECAS, we believe that *Microsystems & Nanoengineering* will become one of the boutique journals in the field. We also believe that the advent of *Microsystems & Nanoengineering* is bound to make a positive contribution to the spread of new knowledge and the development of microsystems and nanotechnology globally.

## Figures and Tables

**Figure 1:**
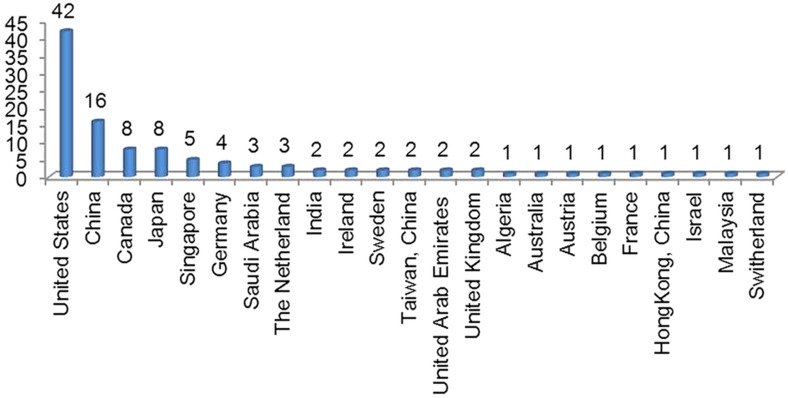
Distribution of submissions among countries/regions.

**Figure 2:**
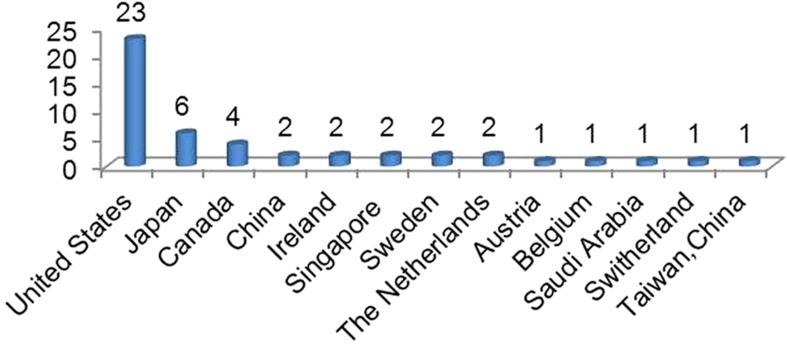
Distribution of accepted papers among countries/regions.

**Figure 3:**
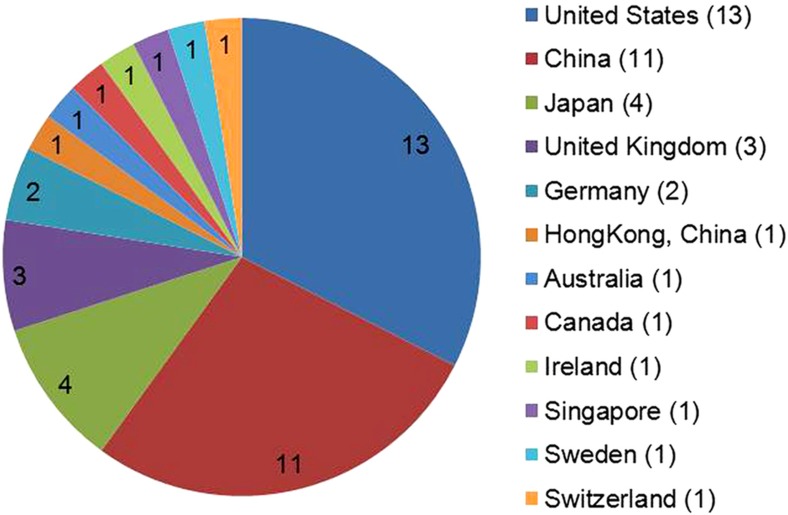
40 editorial board members from 12 countries and regions.

**Figure 4:**
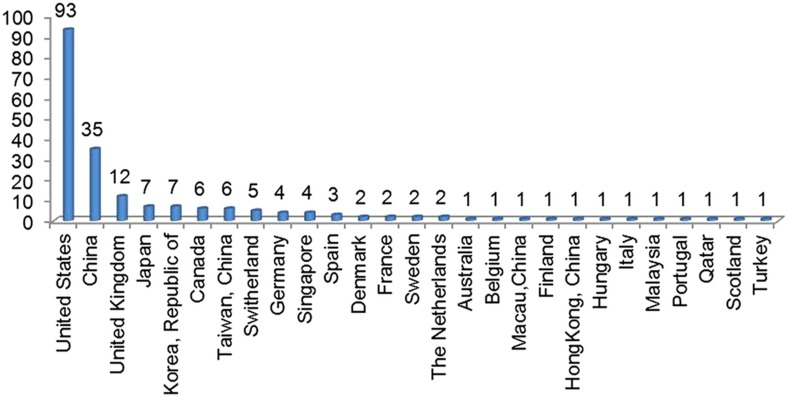
Distribution of reviewers who served for *Microsystems & Nanoengineering*.

